# Research on Vibration Amplitude of Ultra-Precision Aerostatic Motorized Spindle under the Combined Action of Rotor Unbalance and Hydrodynamic Effect

**DOI:** 10.3390/s23010496

**Published:** 2023-01-02

**Authors:** Wenbo Wang, Pengyun Song, Hechun Yu, Guoqing Zhang

**Affiliations:** 1 Faculty of Mechanical and Electrical Engineering, Kunming University of Science and Technology, Kunming 650500, China; 2 School of Mechatronics Engineering, Zhongyuan University of Technology, Zhengzhou 451191, China

**Keywords:** aerostatic motorized spindle, rotor unbalance, equilibrium position, hydrodynamic effect

## Abstract

In the working process of the gas bearings, the unbalanced force of the rotor will increase nonlinearly with the increase in the rotating speed, resulting in an increase in the rotor’s vibration amplitude. On the other hand, with the increase in the rotating speed, the hydrodynamic effect will increase, and the nonlinear increase in the gas film force and stiffness will inhibit the increase in the vibration amplitude. In order to deeply study the influence of the unbalanced force and nonlinear gas film force on the vibration amplitude of the ultra-precision aerostatic motorized spindle, taking the double slit throttling gas bearing as an example, according to the equilibrium equation of the rotor under the combined action of gravity, the gas film force, and the unbalanced force, a calculation program based on the finite difference method for solving the rotor’s equilibrium position is completed. The calculation results show that: the hydrodynamic effect can significantly increase the bearing capacity and cause the change of the static equilibrium position of the rotor, but the offset amplitude of the static equilibrium position of the rotor gradually slows down with the increase in the rotating speed. The hydrodynamic effect improves the stiffness near the static equilibrium position of the rotor, making the rotor vibration track tend to be more “round”. Although the unbalanced force of the rotor increases nonlinearly as the rotating speed increases, the maximum offset between the dynamic equilibrium position and the static equilibrium position of the rotor under the action of the rotating unbalanced force is approximately linear with the rotating speed. Compared with the air supply pressure, the rotor unbalance and rotating speed are the main factors affecting the rotor dynamic equilibrium position offset. This study has a reference role in the in-depth study of the influence of rotating speed and rotor unbalance on the rotor static equilibrium position and dynamic equilibrium position offset, as well as in the design of gas bearings and the prediction of rotor vibration amplitude.

## 1. Introduction

The gas bearing has the characteristics of low friction, high speed, and high precision [1], and it is widely used in the field of precision and ultra-precision machining and measurement [2]. At present, the rotary accuracy of the ultra-precision spindle has entered the nano era. For example, the Moore Nanotech 250UPL ultra-precision single point diamond lathe [3] has a spindle runout of less than 12.5 nm in the full speed range (50–10,000 rpm). All factors affecting the rotary accuracy of the spindle should be fully considered in the design [4]. In precision machining, the optimum process parameters can be obtained via experiments or through the establishment of cutting models [5,6]; however, the rotating accuracy of rotor is usually lack of effective means to further improve after the rotor assembly. Therefore, as an important error source in ultra-precision machining [7], the rotating error of the rotor should be scientifically and reasonably evaluated at the beginning of the design. When evaluating the rotation accuracy of the rotor, the total error motion of the rotor can be regarded as an asynchronous error motion superimposed on the completely repeated synchronous error motion contour [8]. The unbalance force of the rotor is generated by an unbalanced mass during rotation, which is an important source of synchronous error; due to factors such as uneven material, process error, and uneven wear in use, rotor unbalance cannot be avoided. The existence of the unbalanced mass will cause the unbalanced force, which are periodic changes as the rotor rotates, and the frequency is the same as the rotor rotating frequency. The rotor unbalance will cause vibration, damage the bearing surface, shorten the service life, and reduce the rotating accuracy. The dynamic balancing of the rotor is needed during the manufacturing, maintenance, and operation of the rotor system, so that the rotor vibration caused by the unbalanced force of the rotor or the dynamic load acting on the bearing can be reduced to the allowable range. Although the rotor unbalance can reach a small range with the help of the dynamic balancing machine, the vibration of the rotor caused by rotor unbalance still has a huge impact on the ultra-precision rotor, and it is necessary to deeply study the impact of the rotor unbalance on the rotor’s vibration amplitude. For ultra-rotating accuracy occasions, an increase in the rotating speed may lead to the deterioration of the rotary accuracy, for example, when the rotating speed increases to a certain value in ultra-precision turning and flying cutting, the surface roughness tends to increase [9,10]. On the one hand, the rotating speed is restricted by motor power, cutting conditions [11], materials [12,13,14], tool heat dissipation and wear; on the other hand, with the increase in the rotating frequency and the amplitude of unbalanced force, the spindle vibration, tool tip vibration, and guide rail vibration caused by the rotating speed will become more significant. Furthermore, resonance will occur when the frequency of the unbalanced force is close to the natural frequency, and when the rotating speed is close to twice the critical speed, it is easy to produce significant half-frequency whirl and instability. The increase in speed will lead to a sharp rise in the unbalance force of rotor; for the ultra-precision spindle with high requirements of rotary accuracy, the operating speed is often set below the first critical speed when the requirements for its use are met. At this time, the bearing stiffness is far less than the rotor stiffness. The smaller bearing stiffness and rotor imbalance force are not enough to cause significant elastic deformation of the rotor; in the research, a rigid rotor model is often used and the inertia force can be ignored [15].

At present, many scholars have carried out a lot of research on gas bearings. In terms of gas bearing performance analysis, Du et al. [16] studied the load capacity of bearing by opening pressure-equalizing grooves of different structural forms, different positions, and different numbers on the inner surfaces of aerostatic journal bearings through numerical simulation and experimental research. Chen et al. [17] analyzed the stiffnesses of various geometric designs of aerostatic journal bearings under different operating conditions, and analyzed the measures to improve the stiffness. Swanson et al. [18] combined the magnetic bearing and the gas foil bearing to improve the damping, stiffness, and bearing capacity of the bearing system. In general, the research on the load capacity of aerostatic bearing mainly focuses on changing the throttling mode or on opening different spiral grooves or pressure-equalizing grooves in aerostatic bearingm or optimizing the design of the bearing structure, such as the use of the gas magnetic hybrid support bearing, so as to improve the load capacity of the aerostatic bearing. Research on the influence of manufacturing errors on the performance of aerostatic bearings has also been studied, Cui et al. [19,20] studied the influence of manufacturing errors on the running accuracy of the aerostatic porous spindle, Zhang et al. [21] studied the influence of rotor shape errors on rotating accuracy, and quantified the influences of rotor shape errors on its rotating accuracy [22]. In terms of rotordynamic analysis, San Andrés et al. [23] described the measurements of mass flow rate and unbalance response conducted on a solid rigid rotor supported on a pair of porous gas bearings and tilting pad type, and found that the amplitude of rotor response is proportional to the unbalance mass. Liu et al. [24] measured the rotordynamic response of a rotor supported on porous-type gas bearings, and found that the synchronous amplitude increases as the unbalance increases for all of the tested bearing supply pressures. Zhang et al. [25] deduced the mathematical solutions for a five-degrees-of-freedom dynamic model of an aerostatic bearing spindle. Most scholars focus on the critical situation before instability, or the relevant influencing factors before instability [26]. By comparing the previous literature, the research on hydrodynamic effect mainly focuses on bearing capacity and stiffness, and the research on rotor vibration amplitude under the combined actions of rotor unbalance and the hydrodynamic effect lack systematic analysis and research on the rotor trajectory of gas bearings mainly carried out by establishing the dynamic equation and solving the non-homogeneous equation [27]. However, the bearing stiffness and damping are not fixed values with the rotor whirl; using a fixed value will inevitably produce errors, and the gas film force is distributed along the axial direction of the rotor; simplifying the gas bearing into two fulcrums does not conform to the actual situation. Most scholars mainly study the stability of gas bearings at high speed; studies on the rotor rotating accuracy have usually focused on the test measurement of the rotor rotating accuracy and the error separation of the rotor; there are few studies on the influence of the rotor unbalance on the rotor rotating accuracy from the perspective of the rotor equilibrium position [28]. For the ultra-precision aerostatic motorized spindle, the influence of rotor unbalance cannot be ignored. At a certain moment, under the combined action of gas film force, gravity, and unbalanced force, the rotor will deviate from the static equilibrium position, and there is a specific dynamic equilibrium position making the resultant force of rotor zero. Since the direction of the unbalanced force changes periodically with the rotor’s rotation, the dynamic equilibrium position is deviated periodically with the static equilibrium position as the center, which is a closed curve that is related to the unbalanced force of the rotor. At any time, there is a unique dynamic equilibrium position when the magnitude and direction of the unbalanced force is known. As the rotor deviates from the equilibrium position, the resultant force on the rotor is not zero and it acts as an elastic restoring force to make the rotor move to the equilibrium position. When the rotor is in a steady periodic motion, the rotor vibration amplitude can be reflected approximately by solving the rotor dynamic equilibrium position at a certain time. Due to the existing commercial software such as ANSYS Fluent [29] being unable to directly solve the rotor equilibrium position under multiple forces, this paper establishes a simulation program to calculate the rotor static and dynamic equilibrium position, and on this basis, the vibration amplitude of the aerostatic motorized spindle under the combined actions of rotor unbalance and the hydrodynamic effect is studied, and it is different from the previous calculation, which is usually carried by solving the rotor motion differential equation.

## 2. Mathematical Models

### 2.1. Physical Model Structure

**Figure 1:**
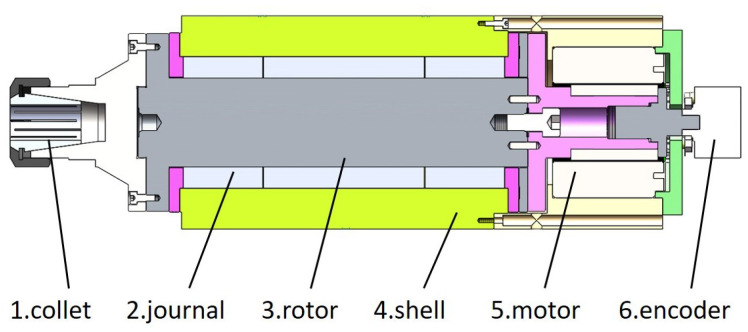
Aerostatic motorized spindle.

**Figure 2:**
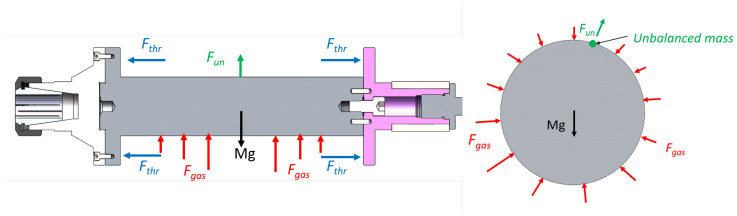
Force model.

The aerostatic motorized spindle used in this study is shown in Figure 1, which is mainly composed of a motor, a journal bearing (journal and rotor), a thrust bearing, and a shell and gas supply system. The force exerted on the rotor during the operating conditions is shown in Figure 2, which can be divided into: the gravity of the rotor and its related accessories Mg, the radial gas film force $F_{gas}$, the axial thrust force $F_{thr}$, and the unbalanced force of rotor $F_{un}$. The unbalanced force is related to the unbalanced mass of the rotor, and its direction changes periodically with the rotor’s rotation. Since the thrust force is far greater than the unbalanced force, it can be approximately considered that the rotor is in a horizontal state; that is, there will be no cone instability in the case of static unbalance, and when ignoring the tilting moment generated by the rotor unbalance, the unbalanced mass of the rotor can be combined into one point. The structural parameters of the motorized spindle are presented in Table 1.

### 2.2. Gas Film Force

By integrating the horizontal and vertical components of the rotor surface pressure, the resultant forces in the X and Y directions can be obtained:

(1) $$F_{x}^{gas}=\int_{0}^{L_{1}}\int_{0}^{2\pi }pr_{1}\sin\beta d\theta dy,$$

(2) $$F_{y}^{gas}=\int_{0}^{L_{1}}\int_{0}^{2\pi }pr_{1}\cos\beta d\theta dy,$$

where $L_{1}$ is the rotor length, *p* is the rotor surface pressure, β is the deviation angle of the rotor, dθ is the angle increment in the circumferential direction of the rotor, dy is the length increment of the rotor, and the rotor surface pressure *p* can be obtained by discretizing the Reynolds equation using the finite difference method.

#### 2.2.1. Boundary Conditions

(1) The whole flow process is regarded as being isothermal, and the viscosity of the gas is assumed to be constant; (2) The Ideal gas and the lubrication gas are Newtonian fluids; (3) The slip effect between bearings is not considered; (4) Ignoring the gas volume force, the gas inertia force and viscous force can be ignored; (5) The influence of bearing surface roughness is ignored.

#### 2.2.2. Reynolds Equation of Gas Lubrication and Hydrodynamic Effect

The general form of the Reynolds equation for compressible gas lubrication under laminar flow is:

(3) $$\frac{\partial }{\partial x}\left(ph^{3}\frac{\partial p}{\partial x}\right)+\frac{\partial }{\partial y}\left(ph^{3}\frac{\partial p}{\partial y}\right)=12\mu \frac{\partial \left(ph\right)}{\partial t}+6U\mu \frac{\partial \left(ph\right)}{\partial x},$$

where μ is the aerodynamic viscosity, *p* is the gas film pressure, *y* is the axial coordinate of the bearing, *x* is the coordinate of the bearing along the rotating direction, *U* is the journal speed, *h* is the gas film gap, and *t* is the time.

The physical meaning of the expansion term at the right end of the equation: $6U\mu p\frac{\partial \left(h\right)}{\partial x}$ is the hydrodynamic effect. When the rotor is eccentric and rotates relative to the journal, the clearance along the direction of rotates gradually decreases, forming a convergence gap. In order to maintain equal flow, the gas flow along the convergence gap will generate positive pressure. The hydrodynamic effect improves the gas film stiffness and thus affects the equilibrium position and the vibration amplitude of the rotor. According to the expansion term, the hydrodynamic effect is related to many factors; the relative rotating speed, air supply pressure, and rotor eccentricity will affect the hydrodynamic effect. Although the hydrodynamic effect cannot be directly described using specific values, it can be studied through the change of bearing capacity and equilibrium position.

According to the above assumptions, the equation can be simplified into the dimensionless form under the steady-state conditions:

(4) $$\frac{\partial }{\partial \bar{x}}\left({\bar{h}}^{3}\frac{\partial {\bar{P}}^{2}}{\partial \bar{x}}\right)+\frac{\partial }{\partial \bar{y}}\left({\bar{h}}^{3}\frac{\partial {\bar{P}}^{2}}{\partial \bar{y}}\right)=2\Lambda \frac{\partial \left(\bar{P}\bar{h}\right)}{\partial \bar{x}},$$

where

(5) $$\bar{x}=\frac{x}{r},\bar{y}=\frac{z}{r},\bar{P}=\frac{p}{p_{a}},\bar{h}=\frac{h}{C_{1}},U=r\omega ,\Lambda =\frac{6\mu \omega R_{1}^{2}}{p_{a}c^{2}},$$

**Figure 3:**
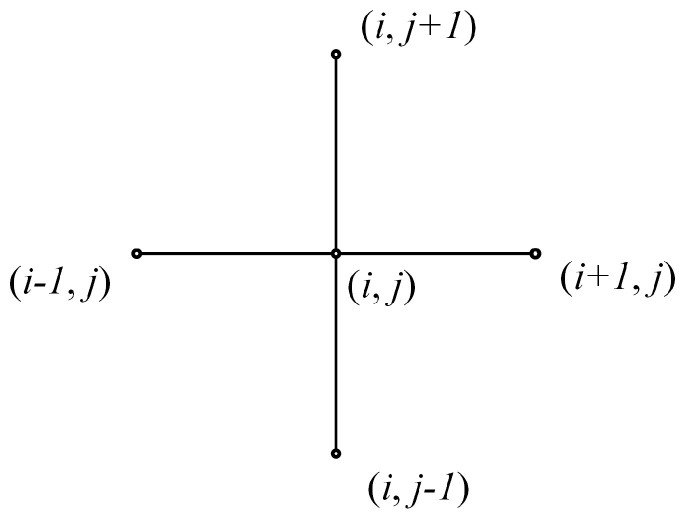
Five-point difference method.

*r* is the rotor radius, $p_{a}$ is the environmental pressure, *c* is the mean gas film gap, ω is the angular velocity, and $R_{1}$ is the journal radius. The five-point difference method is shown in Figure 3, and Equations (6)–(8) are the finite difference equations.

(6) $$\frac{\partial }{\partial \bar{x}}{\left({\bar{h}}^{3}\frac{\partial {\bar{P}}^{2}}{\partial \bar{x}}\right)}_{i,j}=\frac{{\bar{h}}_{i+0.5,j}^{3}\left({\bar{P}}_{i+1,j}^{2}-{\bar{P}}_{i,j}^{2}\right)}{\Delta {\bar{x}}^{2}}-\frac{{\bar{h}}_{i-0.5,j}^{3}\left({\bar{P}}_{i,j}^{2}-{\bar{P}}_{i-1,j}^{2}\right)}{\Delta {\bar{x}}^{2}},$$

(7) $$\frac{\partial }{\partial \bar{y}}{\left({\bar{h}}^{3}\frac{\partial {\bar{P}}^{2}}{\partial \bar{y}}\right)}_{i,j}=\frac{{\bar{h}}_{i,j+0.5}^{3}\left({\bar{P}}_{i,j+1}^{2}-{\bar{P}}_{i,j}^{2}\right)}{\Delta {\bar{y}}^{2}}-\frac{{\bar{h}}_{i,j-0.5}^{3}\left({\bar{P}}_{i,j}^{2}-{\bar{P}}_{i,j-1}^{2}\right)}{\Delta {\bar{y}}^{2}},$$

(8) $$\frac{\partial }{\partial \bar{x}}{\left(\bar{P}\bar{h}\right)}_{i,j}=\frac{{\bar{P}}_{i,j+1}{\bar{h}}_{i,j+1}-{\bar{P}}_{i,j-1}{\bar{h}}_{i,j-1}}{2\Delta \bar{x}},$$

Equations (4)–(8) can be converted into the difference expression of the Reynolds equation, and the pressure at point (i, j) can be obtained:

(9) $${\bar{P}}_{i,j}=\sqrt{\frac{{\bar{A}}_{1}{\bar{P}}_{i,j+1}^{2}+{\bar{A}}_{2}{\bar{P}}_{i,j-1}^{2}+{\bar{A}}_{3}{\bar{P}}_{i+1,j}^{2}+{\bar{A}}_{4}{\bar{P}}_{i-1,j}^{2}-{\bar{A}}_{5}\left({\bar{P}}_{i,j+1}^{}{\bar{h}}_{i,j+1}^{}-{\bar{P}}_{i,j-1}^{}{\bar{h}}_{i,j-1}^{}\right)}{{\bar{A}}_{1}+{\bar{A}}_{2}+{\bar{A}}_{3}+{\bar{A}}_{4}}},$$

where

$${\bar{A}}_{1}=\frac{{\left({\bar{h}}_{i,j}+{\bar{h}}_{i,j+1}\right)}^{3}}{2^{4}\cdot \Delta {\bar{x}}^{2}},{\bar{A}}_{2}=\frac{{\left({\bar{h}}_{i,j}+{\bar{h}}_{i,j-1}\right)}^{3}}{2^{4}\cdot \Delta {\bar{x}}^{2}},{\bar{A}}_{3}=\frac{{\left({\bar{h}}_{i,j}+{\bar{h}}_{i+1,j}\right)}^{3}}{2^{4}\cdot \Delta {\bar{y}}^{2}},$$

$${\bar{A}}_{4}=\frac{{\left({\bar{h}}_{i,j}+{\bar{h}}_{i-1,j}\right)}^{3}}{2^{4}\cdot \Delta {\bar{y}}^{2}},{\bar{A}}_{5}=\frac{3\mu \omega R^{2}}{Pa\cdot h_{{}_{0}}^{2}\Delta x}$$

#### 2.2.3. Calculation of Gas Film Flow

According to the law of mass conservation, the mass of flowing gas from the slit is equal to the mass flowing from the bearing gap, so the gas pressure $P_{d}$ at each point at the junction of the slit and the bearing gap can be obtained. According to the N-S equation, the velocity distribution of the gas film in the slit and bearing gap can be solved simply, and the mass of flowing gas can be obtained via integration. The mass of the flowing gas through the slit and the bearing gap are:

(10) min=Ps2−Pd2πH312mRTln(r1r1r2r2),

(11) $$m_{out}=\frac{1}{24\mu RT}\sum \left[\left(p_{\left(d,j\right)}^{2}-p_{\left(d+1,j\right)}^{2}\right)h_{\left(d+\frac{1}{2},j\right)}^{3}\frac{d\phi }{dy}+\left(p_{\left(d,j\right)}^{2}-p_{\left(d-1,j\right)}^{2}\right)h_{\left(d-\frac{1}{2},j\right)}^{3}\frac{d\phi }{dy}\right],$$

where *R* is the gas constant, *H* is the throttle slit width, *T* is the absolute temperature, $r_{1}$ is the radius of the outer slit, and $r_{2}$ is the radius of the inner slit. Ignoring the gas diffusion flow at the junction of the slit and the bearing gap, and without considering the influence of circumferential flow, the mass flow from the junction of the slit and the bearing gap is equal to the outflow mass of the rotor, i.e., $m_{in}=m_{out}$, and the pressure at each point at the junction of the slit and the bearing gap can be obtained:

(12) $$p_{d,j}^{2}=\frac{p_{s}^{2}H^{3}\Delta y+\left(p_{d+1,j}^{2}h_{d+\frac{1}{2},j}^{3}+p_{d-1,j}^{2}h_{d-\frac{1}{2},j}^{3}\right)R\ln\left(r_{1}/r_{2}\right)}{H^{3}\Delta y+\left(h_{d+\frac{1}{2},j}^{3}+h_{d-\frac{1}{2},j}^{3}\right)R\ln\left(r_{1}/r_{2}\right)}.$$

### 2.3. Permissible Unbalanced Mass and Unbalanced Force of Rotor

The equation for calculating the permissible unbalanced mass during the dynamic balancing is:

(13) $$m_{per}=M\times G\times \frac{60}{2\pi \times r\times n}\times 10^{3},$$

where: *M* is the rotor mass, *G* is the grade of balancing of the rotor, *r* is the balancing radius of the rotor, and *n* is the balancing speed of the rotor. The unbalanced force of the rotor generated via the permissible unbalanced mass is:

(14) $$F_{un}=m_{per}\times r\times {\left(\frac{2\pi n}{60}\right)}^{2}.$$

### 2.4. Equilibrium Equation and Calculation Flow

As the amplitude of the thrust force is far greater than the unbalanced force of the rotor, it is approximately considered that the overturning moment generated by the unbalanced force is not enough to change the horizontal state of the rotor. The pressure of each point on the rotor surface $P_{i,j}$ is related to the deviation angle β and the eccentricity *e* of the rotor. In addition to the gas film forces, the rotor is also subjected to gravity Mg. The static equilibrium position of the rotor should meet the following conditions:

(15) $$W_{x}=\int_{0}^{L_{1}}\int_{0}^{2\pi }pr_{1}\sin\beta d\theta dy=0,$$

(16) $$W_{y}=\int_{0}^{L_{1}}\int_{0}^{2\pi }pr_{1}\cos\beta d\theta dy-Mg=0.$$

The discrete forms of the static equilibrium equations are:

(17) $$W_{x}=\sum p_{i,j}\left(\beta +\delta_{\beta },e+\delta_{e}\right)r_{1}\Delta \beta \Delta L_{1}sin\left(\beta +\delta_{\beta }\right)=0,$$

(18) $$W_{y}=\sum p_{i,j}\left(\beta +\delta_{\beta },e+\delta_{e}\right)r_{1}\Delta \beta \Delta L_{1}\cos\left(\beta +\delta_{\beta }\right)-Mg=0,$$

where: $\delta_{\beta }$ is the amount of deviation angle change, $\delta_{e}$ is the amount of eccentricity change, $W_{x}$ is the resultant force along the X direction, $W_{y}$ is the resultant force along the Y direction, and Mg is the gravity of the rotor.

The rotor unbalance cannot be avoided, and when the unbalanced force $F_{un}$ is not zero, the rotor whirl will occurs at the rotating frequency centered on the static equilibrium position. The amplitude of the unbalanced force $F_{un}$ is related to the rotor speed *n*, and the direction changes periodically with the rotor’s rotation. The dynamic equilibrium position of the rotor at any time under the action of an unbalanced force should meet the following conditions:

(19) $$W_{x}=\int_{0}^{L_{1}}\int_{0}^{2\pi }pr_{1}\sin\beta d\theta dy+F_{un}\cos\gamma =0,$$

(20) $$W_{y}=\int_{0}^{L_{1}}\int_{0}^{2\pi }pr_{1}\cos\beta d\theta dy-Mg-F_{un}\sin\gamma =0.$$

The discrete forms of the dynamic equilibrium equations are:

(21) $$W_{x}=\sum p_{i,j}\left(\beta +\delta_{\beta },e+\delta_{e}\right)r_{1}\Delta \beta \Delta L_{1}sin\left(\beta +\delta_{\beta }\right)+F_{un}\cos\gamma =0,$$

(22) $$W_{y}=\sum p_{i,j}\left(\beta +\delta_{\beta },e+\delta_{e}\right)r_{1}\Delta \beta \Delta L_{1}\cos\left(\beta +\delta_{\beta }\right)-Mg-F_{un}\cos\gamma =0,$$

where: γ is the angle of the unbalanced force with the vertical direction. The offset from the dynamic equilibrium position to the static equilibrium position can be obtained as:

(23) $$R=\sqrt{{\left[\left(e+\delta_{e}\right)\cos\left(\beta +\delta_{\beta }\right)-e\cos\beta \right]}^{2}+{\left[\left(e+\delta_{e}\right)\sin\left(\beta +\delta_{\beta }\right)-e\sin\beta \right]}^{2}}.$$

**Figure 4:**
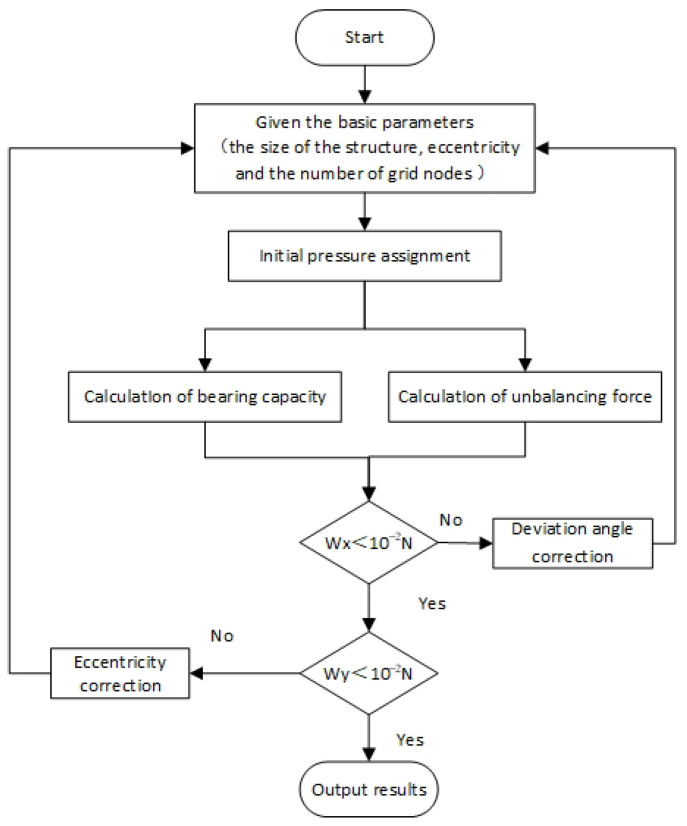
Calculation flow chart.

The model is calculated based on the Reynolds equation and the finite difference method. First, the film thickness and pressure distribution are obtained. Second, the bearing capacity, eccentricity, and deviation angle are obtained. Third, judging whether the resultant force in the horizontal and vertical directions is zero according to the equilibrium equations, when the resultant force is not zero, the eccentricity and deflection angle are corrected again until $W_{x}<10^{-2}$ N and $W_{y}<10^{-2}$ N, then the static equilibrium position and dynamic equilibrium position are obtained, respectively. Finally, the offset between the dynamic equilibrium position and the static equilibrium position is obtained. Figure 4 is the calculation flow chart.

## 3. Results and Discussion

### 3.1. Correctness Verification of Calculation

**Figure 5:**
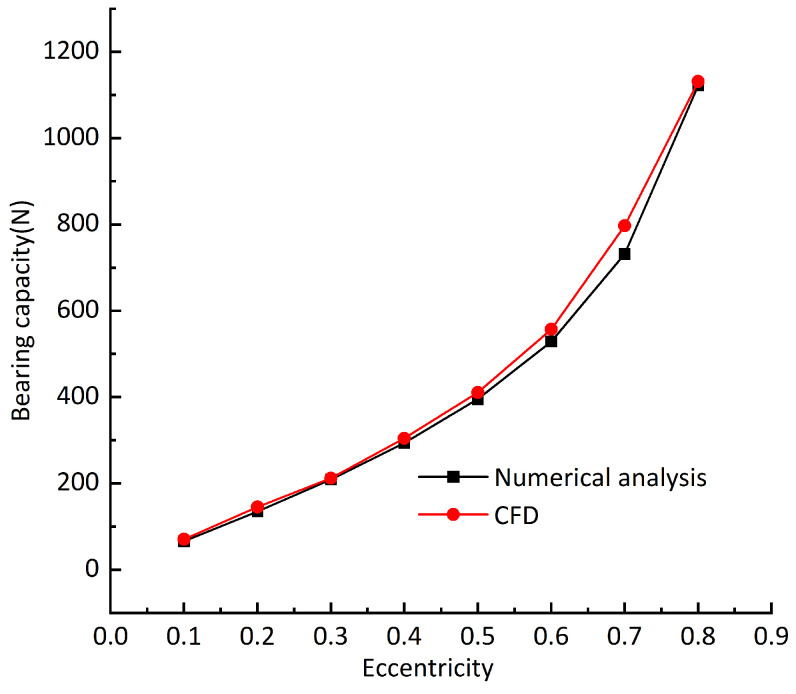
Bearing capacity under two methods.

As the research in this paper has not been verified by experiments, the calculation results are compared with the ANSYS Fluent [29] software. Figure 5 shows the comparison results of the bearing capacity; the air supply pressure is 0.5 Mpa and the rotating speed is 10,000 rpm. It can be seen from the figure that the bearing capacity increases with the increase in eccentricity; the bearing capacity obtained using the two calculation methods is slightly different, and the growth trend is approximately the same. The grid division and different parameter settings will make the calculation results slightly different, so it can be considered that the method used in this paper is in line with the reality.

### 3.2. Influence of Hydrodynamic Effect on Bearing Capacity

**Figure 6:**
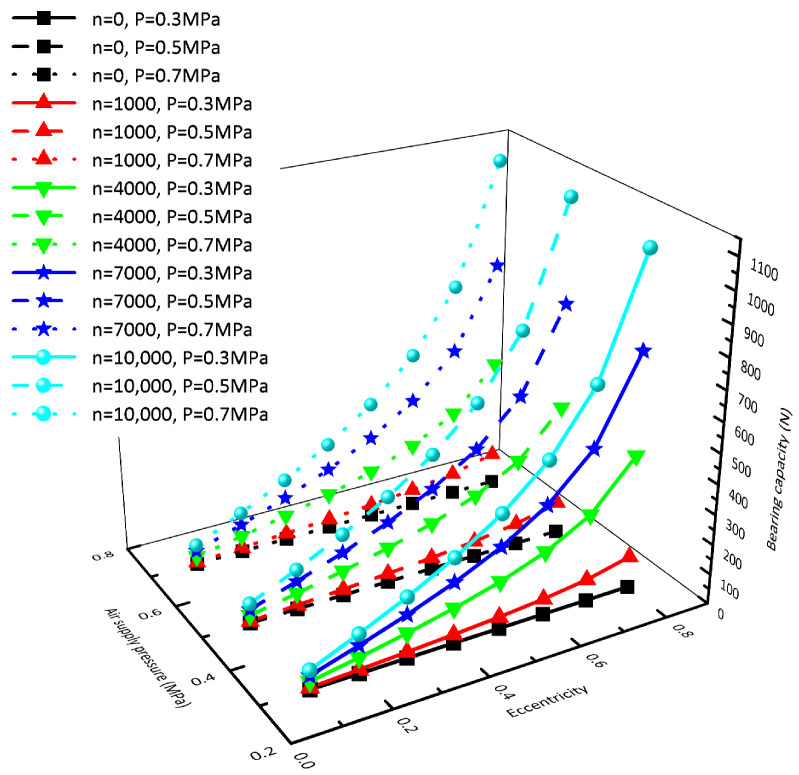
Influence of eccentricity on bearing capacity.

**Figure 7:**
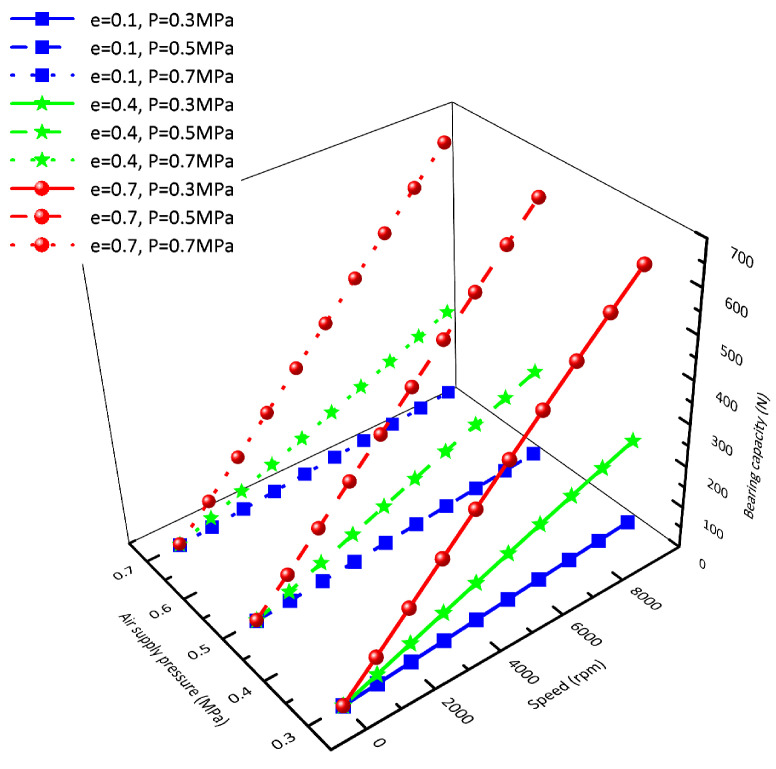
Influence of rotating speed on bearing capacity.

In order to research the influence of the hydrodynamic effect on the bearing capacity of the rotor, two methods of fixed speed and fixed eccentricity are used. Figure 6 shows the bearing capacity obtained by changing the air supply pressure and eccentricity when the rotating speed is *n* = 0 rpm (black line), *n* = 1000 rpm (red line), *n* = 4000 rpm (green line), *n* = 7000 rpm (blue line), and *n* = 10,000 rpm (cyan line), respectively. By comparing the five rotating speeds, we can see that: at the same rotating speed, the air supply pressure has little effect on the bearing capacity, and the bearing capacity increases nonlinearly with the increase in eccentricity; the higher the rotating speed, the more significant the influence of eccentricity on the bearing capacity. Figure 7 shows the bearing capacity obtained by changing the air supply pressure and rotating speed when the eccentricity is taken as e = 0.1 (blue line), e = 0.4 (green line), and e = 0.7 (red line), respectively. It can be seen from the comparison of the three kinds of eccentricity that: the air supply pressure has little influence on the rotor bearing capacity under the same eccentricity, and the bearing capacity increases linearly with the increase in rotating speed. The greater the eccentricity, the more significant the influence of rotating speed on the bearing capacity.

### 3.3. Influence of the Hydrodynamic Effect on the Static Equilibrium Position

**Figure 8:**
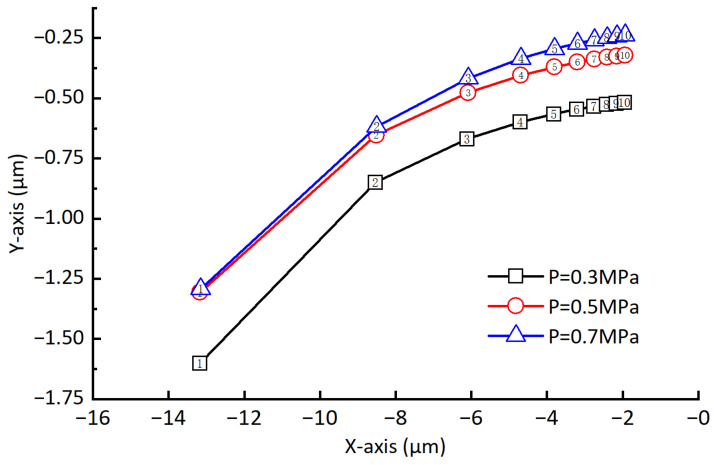
Static equilibrium position under different pressures and rotating speeds.

**Figure 9:**
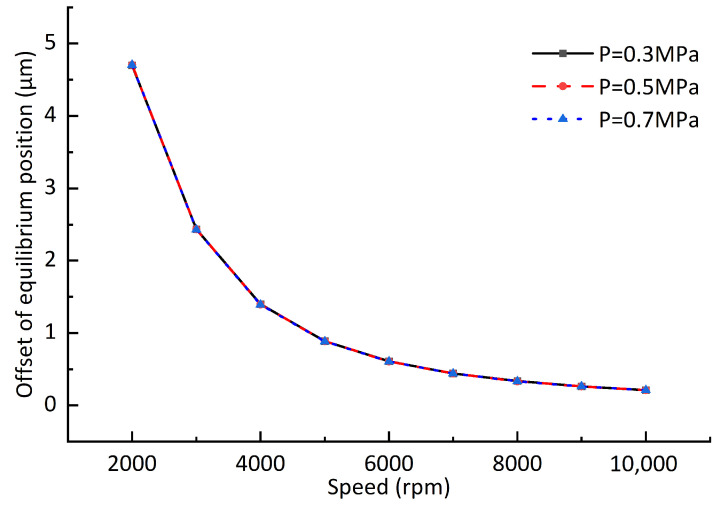
Offsets of static equilibrium position under different pressures and rotating speeds.

In order to observe the difference more clearly, we project all the curves onto the same vertical plane; when the rotor unbalance is not considered, the changes of the static equilibrium position of the rotor at different rotating speeds are shown in Figure 8. The journal center is the origin of the Cartesian coordinate system. It can be seen from the figure that the static equilibrium position of the rotor gradually moves toward the journal center with the increase in the rotating speed, and the amount of offset gradually decreases with the increase in the rotating speed (the number in the figure represents 1000 times the rotating speed). The static equilibrium position of the rotor is different under different air supply pressures. The higher the pressure, the closer the rotor is to the journal center. Compared with the air supply pressure, the rotating speed has a greater impact on the static equilibrium position. When the rotating speed is greater than 4000 rpm, the static equilibrium position offset becomes slower. Figure 9 shows the static equilibrium position offsets under different air supply pressures and rotating speeds. It can be seen from the figure that although the static equilibrium position is different under different air supply pressures, the offsets of static equilibrium position caused by the speed increase is almost the same. Compared with the air supply pressure, the rotating speed has a greater impact on the static equilibrium position’s offset. Under different air supply pressures, the offset of the static equilibrium position caused by increasing the rotor rotating speed is approximately the same. When the rotating speed increases from 1000 rpm to 2000 rpm, the offset of the static equilibrium position is about 4.7 μm. As the rotating speed continues to increase; for example, when the rotating speed increases from 9000 rpm to 10,000 rpm, the offset of the static balance position is only about 0.21 μm.

**Figure 10:**
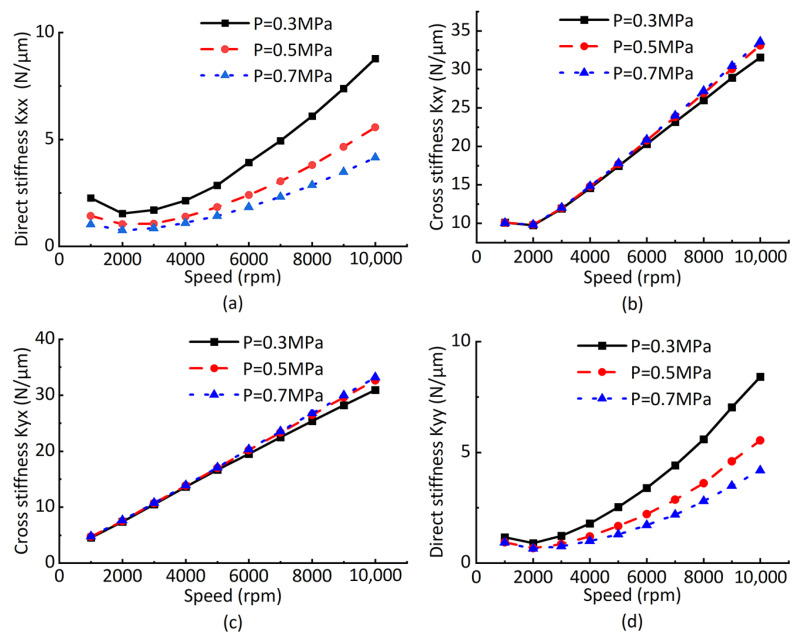
Bearing stiffness curve at different rotor equilibria. (**a**) Direct stiffness in the X direction. (**b**) Cross stiffness in the X direction. (**c**) Cross stiffness in the Y direction. (**d**) Direct stiffness in the X direction.

Move the static equilibrium positions 0.1 μm along X and Y direction, respectively, then calculate the corresponding main stiffness and cross stiffness, as shown in Figure 10. It can be seen from the figure that: with the increase in rotating speed, the main stiffness and cross stiffness at the static equilibrium position increase at the same time, and that the cross stiffness is greater than the main stiffness. This is because the deviation angle exists, and the maximum circumferential pressure of the rotor is near the minimum air gap. Different air supply pressures have different effects on the stiffness at the static equilibrium position. Increasing the air supply pressure will reduce the main stiffnesses in the X and Y directions at the static equilibrium position, while the cross stiffnesses in the X and Y directions increase. It can be seen from the figure that the increase in the rotating speed enhances the hydrodynamic effect and increases the bearing stiffness. The increase in bearing stiffness further leads to the increase in the gas film force at the original static equilibrium position; thus, the resultant force is greater than zero. Under the action of the gas film force, the minimum gas film gap of the rotor increases, and the increase in the minimum gas film gap causes the decrease in the gas film force, finally reaching a new static equilibrium position, which explains the phenomenon where the rotor gradually moves towards the journal center with the increase in rotating speed.

### 3.4. Research on Dynamic Equilibrium Position under the Combined Actions of Rotor Unbalance and Hydrodynamic Effect

#### 3.4.1. Dynamic Equilibrium Position

**Figure 11:**
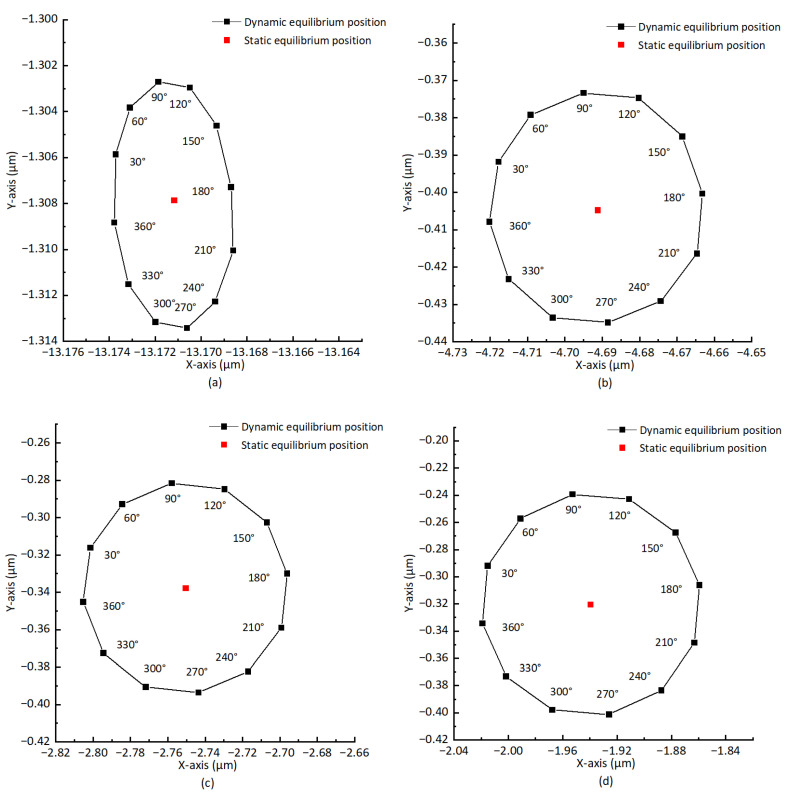
Static and dynamic equilibrium positions at different rotating speeds. (**a**) *n* = 1000 rpm. (**b**) *n* = 4000 rpm. (**c**) *n* = 7000 rpm. (**d**) *n* = 10,000 rpm.

Take the working condition: the air supply pressure *P* = 0.5 Mpa and the grade of balancing *G* = 0.4 as an example, the unbalance force is added every 30${}^{\circ }$ on the rotor surface in turn, and the new dynamic equilibrium position is obtained. The distance between the new dynamic equilibrium position and the static equilibrium position is taken as the offset caused by the unbalanced force. It can be seen from Figure 11 that the maximum offsets of the dynamic equilibrium position are 0.0056 μm, 0.032 μm, 0.057 μm, and 0.083 μm, respectively, corresponding to the rotating speeds *n* = 1000 rpm, *n* = 4000 rpm, *n* = 7000 rpm, and *n* = 10,000 rpm. Although the maximum offset of the dynamic equilibrium position increases with the rotation speed, the increased hydrodynamic effect makes the offset in each direction tend to be the same, so that the increase in the rotation speed makes the trajectory composed of the rotor dynamic equilibrium position more “round”. The maximum offsets of subfigures b, c, and d are all in the 120${}^{\circ }$ direction; this is because the positive stiffness is less than the negative stiffness along the X and Y directions, which also causes the trajectory composed of the dynamic equilibrium positions to be approximately elliptical, and this is consistent with the orbit of rotor center measured from experiments in the past. The direction of maximum offset in figure a is 270${}^{\circ }$; the offset is close to 0.0050 μm in the 120${}^{\circ }$ direction, and the possible reason is a calculation error, as the unbalanced force is too small at a low rotating speed.

**Figure 12:**
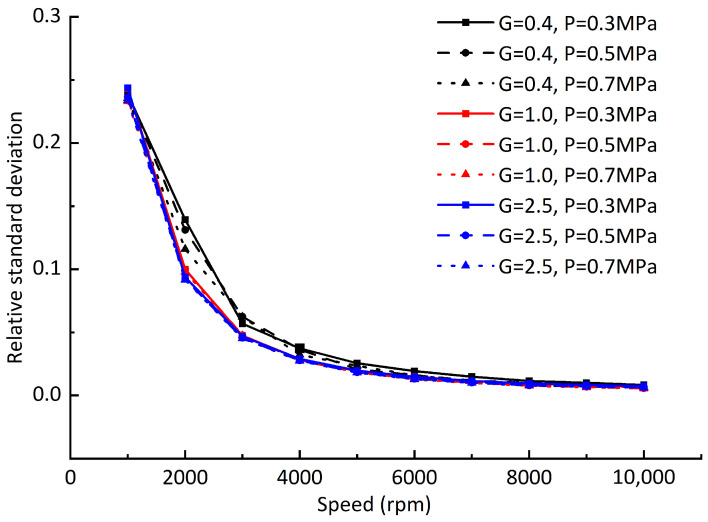
Relative standard deviation of dynamic equilibrium position offsets at different rotating speeds.

**Figure 13:**
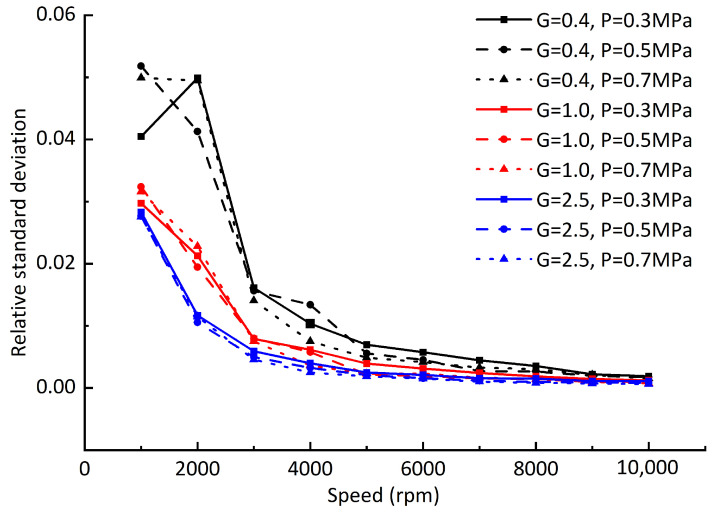
Relative standard deviation of dynamic equilibrium position offsets (gas film gap $C_{1}$ = 10 μm).

**Figure 14:**
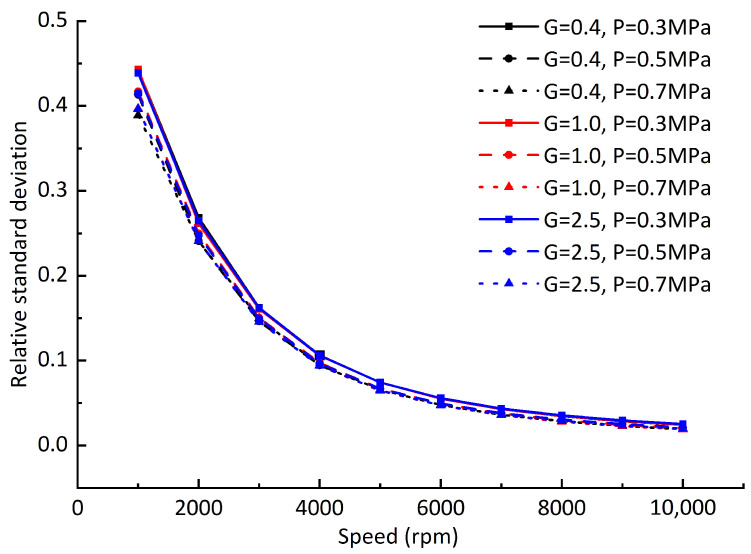
Relative standard deviation of dynamic equilibrium position offsets (adding load *F* = 65 N).

Due to the difference between the offset values of the dynamic equilibrium positions and static equilibrium positions at different rotating speeds, in order to further analyze the difference of the offset values, the relative standard deviation of dynamic equilibrium position and static balance offset is defined by referring to the concept of relative standard deviation in data statistics and analysis:

(24) $$Rsd=\frac{\sqrt{\frac{\sum {}_{i}^{n}{\left(r_{i}-\bar{r}\right)}^{2}}{n}}}{\bar{r}},$$

where $r_{i}$ is the offset of each dynamic equilibrium position from the static equilibrium position, and $\bar{r}$ is the average value of the offset of all dynamic positions from the static equilibrium position. Figure 12 shows the relative standard deviation of the dynamic equilibrium position and the static equilibrium position under different dynamic balancing grades and air supply pressures when the gas film gap is 20 μm. Figure 13 differs from Figure 12 in that the gas film gap is 10 μm, and Figure 14 differs from Figure 12 in that the vertical downward load of 65 N is added. By comparing Figure 12, Figure 13 and Figure 14, it can be seen that with the increase in rotating speed, although the offset between the rotor dynamic equilibrium position and the static equilibrium position increases, the numerical difference of each offset tends to decrease; that is, the trajectory composed of the rotor dynamic equilibrium position becomes more “round”. It can be concluded that the hydrodynamic effect caused by the increase in rotating speed will reduce the difference of the gas film stiffness values at each point around the static equilibrium position. Compared with the gas supply pressure, reducing the film thickness can significantly improve the film stiffness and reduce the offset difference between the dynamic equilibrium position and the static equilibrium position.

#### 3.4.2. The Influence of Rotor Unbalance on the Maximum Offset of Dynamic Equilibrium Position

**Figure 15:**
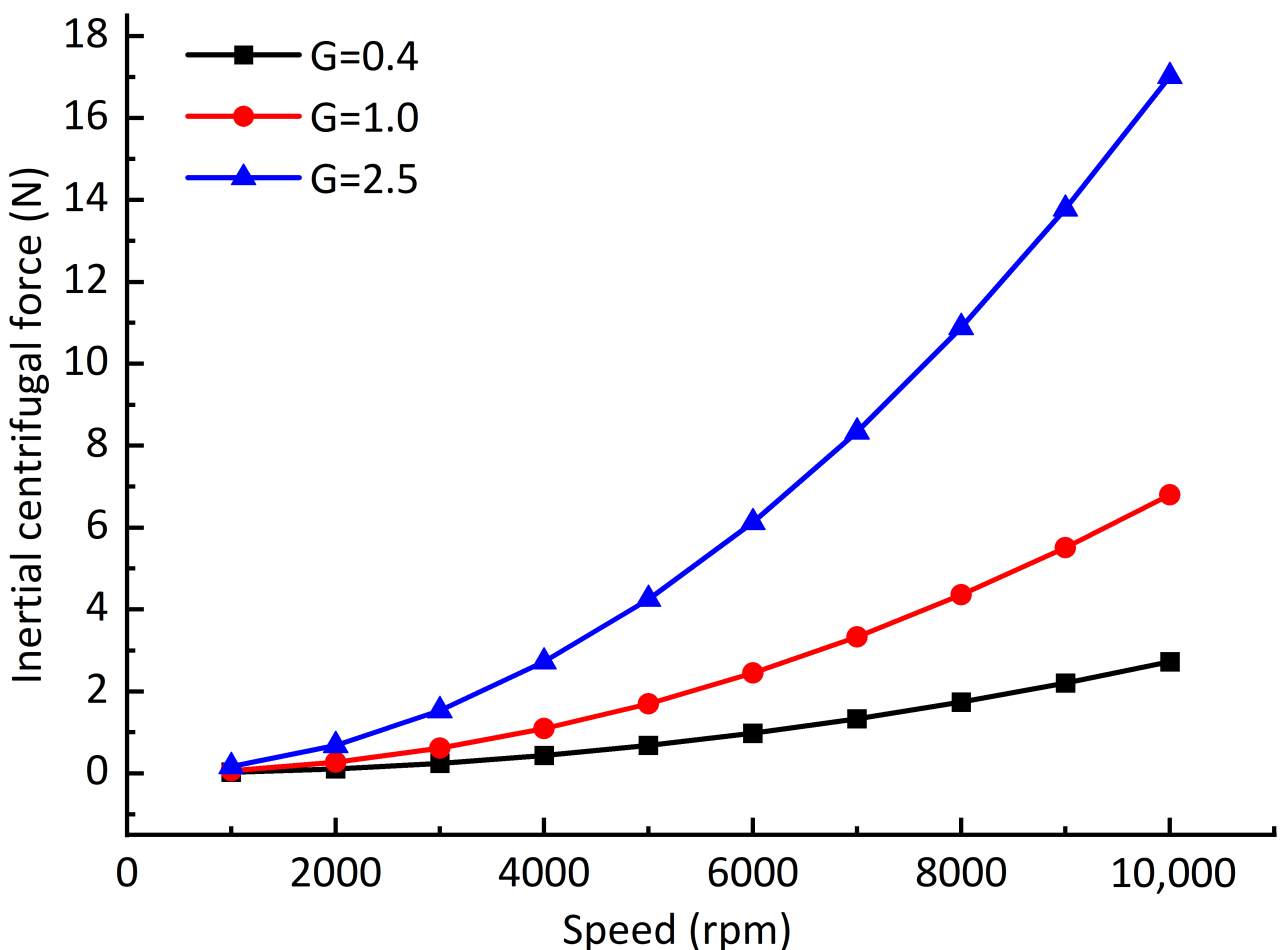
Unbalanced force at different dynamic balancing grades.

**Figure 16:**
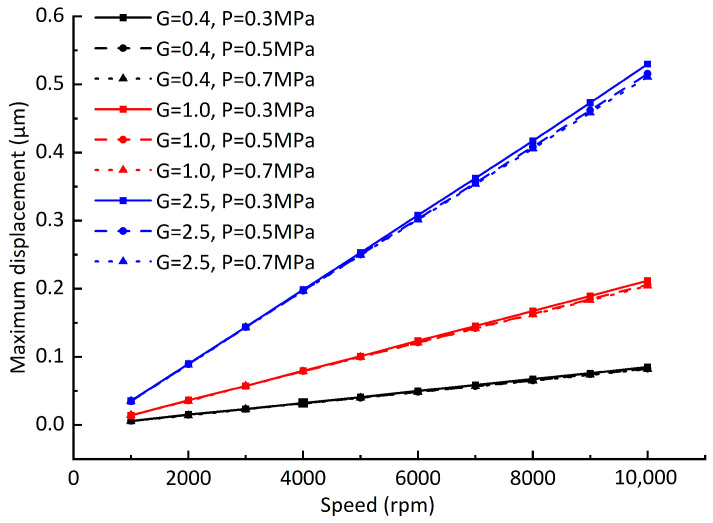
Influence of rotating speed on rotor dynamic equilibrium position offset.

Assuming that the balancing speed *n* = 10,000 rpm, the balancing radius *r* = 25 mm, and when the balancing grades *G* = 0.4, *G* = 1, and *G* = 2.5, the permissible unbalanced masses are *m* = 0.0993 g, *m* = 0.2483 g, and *m* = 0.6207 g, respectively, the maximum unbalanced force caused by the permissible unbalanced mass is shown in Figure 15. The maximum offsets of dynamic equilibrium position caused by unbalanced force under different dynamic balancing grades, different rotating speeds, and different air supply pressures is shown in Figure 16. It can be seen from the comparison of the two figures that: although the unbalanced force changes nonlinearly, the maximum offsets of the dynamic equilibrium position caused by the unbalanced force changes linearly with the rotating speed. The reason for is that the enhancement of the hydrodynamic effect restrains the unbalanced force, but it cannot be completely eliminated. In addition, different air supply pressures have little effect on the offset of the dynamic equilibrium position. From Figure 16, it can be seen that the maximum offset of the dynamic equilibrium position is approximately proportional to the unbalanced mass, which is consistent with San Andrés’ experimental results [23].

**Figure 17:**
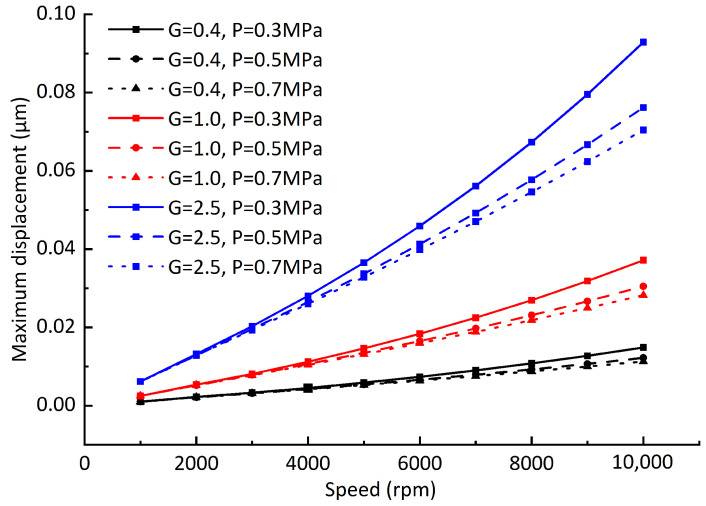
Influence of rotating speed on rotor dynamic equilibrium position offset (gas film gap $C_{1}$ = 10 μm).

**Figure 18:**
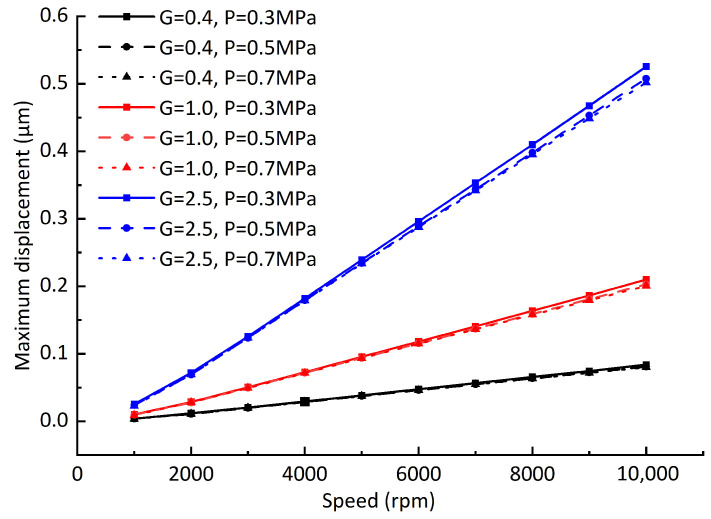
Influence of rotating speed on rotor dynamic equilibrium position offset (adding load *F* = 65 N).

In order to verify the applicability of the conclusions, the gas film gap is changed to 10 μm (Figure 17), the vertical load 65 N is added (Figure 18), and the influences of different dynamic balancing grades on the offsets of the dynamic equilibrium position are recalculated. It can be seen from Figure 17 that the maximum offsets of the dynamic equilibrium position caused by the unbalanced force decreases significantly after the gas film gap decreases. With the increase in the unbalanced mass, the influence of the air supply pressure on the maximum offsets of the dynamic equilibrium position increases. When the air supply pressure is 0.5 MPa and 0.7 Mpa, the maximum offsets of the dynamic equilibrium position are still approximately linear with the rotating speed. Therefore, when the gas film gap is small, increasing the air supply pressure helps to reduce the maximum offsets of the dynamic equilibrium position. When the vertical downward load is added, the rotor eccentricity increases under the action of gravity, the gas film force, and the vertical downward load. It can be seen from Figure 18 that the maximum offsets of the dynamic equilibrium position remain linear with the rotating speed when the rotor eccentricity is increased. Through the above analysis, it can be concluded that the maximum offsets of the dynamic equilibrium position caused by rotor unbalance are approximately linear with the rotating speed. Using this rule, the maximum vibration amplitude of the rotor at some certain speeds can be measured, and the maximum amplitude of the rotor at other speeds can be approximately predicted using the linear relationship.

## 4. Conclusions

1. The static equilibrium position gradually moves towards the center of the journal with the increase in rotating speed, which causes the offset of the rotor whirl track. The rotating speed has a great impact on the static equilibrium position. With the increase in rotating speed, the offset amplitude of the static equilibrium position will gradually decrease. The static equilibrium position is different when the air supply pressures are different, which cannot be ignored in ultra-precision machining and measurement.
2. The difference between horizontal stiffness and vertical stiffness is the reason for why the whirl track of the rotor is approximately elliptical, and for why the direction of the maximum offset points obliquely above. The enhancement of the hydrodynamic effect will make the curve formed by the rotor’s dynamic equilibrium position more “round”.
3. The air supply pressure has little effect on the rotor vibration amplitude, while the rotating speed will have a significant effect on the rotor vibration amplitude. Although the unbalanced force increases nonlinearly with the rotating speed, the hydrodynamic effect caused by the rotating speed increase can weaken the influence of the unbalanced force to a certain extent. The maximum vibration amplitude of the rotor increases approximately linearly with the increase in rotating speed. Reducing the rotor unbalance as much as possible can effectively reduce the maximum vibration amplitude of the rotor. The maximum vibration amplitude of the rotor is approximately proportional to the rotor unbalance when at the same rotation speed and air supply pressure. According to the linear relationship, the maximum vibration amplitude of the rotor can be approximately predicted when the rotation speed or rotor unbalance changes.

## Figures and Tables

**Table 1:** Structural parameters of motorized spindle.

Parameter	Value
Journal radius $R_{1}$ (mm)	25
Journal length $L_{1}$ (mm)	160
Throttle slit width *H* (μm)	80
Slit depth *Y* (mm)	20
Mean gas film gap ($C_{1}$) (μm)	20
Environment pressure ($P_{a}$) (MPa)	0.1
Slit distance $L_{2}$ (mm)	80

## Data Availability

Not applicable.

## References

[1] Gao Q., Chen W., Lu L., Huo D., Cheng K. (2019). Aerostatic bearings design and analysis with the application to precision engineering: State-of-the-art and future perspectives. Tribol. Int..

[2] Karpat Y. (2019). Influence of diamond tool chamfer angle on surface integrity in ultra-precision turning of singe crystal silicon. Int. J. Adv. Manuf. Technol..

[3] Nanotech 250UPL V2 Ultra Precision Lathe. https://nanotechsys.com/250-upl/.

[4] Zhang S., To S., Zhang G., Zhu Z. (2015). A review of machine-tool vibration and its influence upon surface generation in ultra-precision machining. Int. J. Mach. Tools Manuf..

[5] Wojciechowski S., Mrozek K. (2017). Mechanical and technological aspects of micro ball end milling with various tool inclinations. Int. J. Mech. Sci..

[6] Wojciechowski S., Maruda R.W., Krolczyk G.M., Niesłony P. (2018). Application of signal to noise ratio and grey relational analysis to minimize forces and vibrations during precise ball end milling. Precis. Eng..

[7] Tu J.F., Bossmanns B., Hung S.C. (1997). Modeling and error analysis for assessing spindle radial error motions. Precis. Eng..

[8] (2006). Test Code for Machine Tools: Part 7: Geometric Accuracy of Axes of Rotation.

[9] Bi G., Sun Z., Zhang J., Wang Z., An C. (2015). Experiment on main factors affecting surface roughness in ultra-precision fly cutting. Opt. Precis. Eng..

[10] Xiong R., Liu X., Xiong Z., Zhang S., Zhao L. (2020). Experimental study on surface morphology of ultra-precision turning. Manuf. Technol. Mach. Tool.

[11] Chen X., Xu J., Fang H., Tian R. (2017). Influence of cutting parameters on the ductile-brittle transition of single-crystal calcium fluoride during ultra-precision cutting. Int. J. Adv. Manuf. Technol..

[12] Sun Y., Chen W., Liang Y., An C., Chen G., Su H. (2015). Dynamic error budget analysis of an ultraprecision flycutting machine tool. Int. J. Adv. Manuf. Technol..

[13] Cheung C.F., To S., Lee W.B. (2002). Anisotropy of surface roughness in diamond turning of brittle single crystals. Mater. Manuf. Process..

[14] To S., Cheung C.F., Lee W.B. (2001). Influence of material swelling on surface roughness in diamond turning of single crystals. Mater. Sci. Technol..

[15] Liu T. (1990). Aerostatic Gas Lubrication.

[16] Du J., Zhang G., Liu D. (2012). Influences of pressure-equalizing groove on the load capacity of externally pressurized gas journal bearings. Jixie Gongcheng Xuebao (Chin. J. Mech. Eng.).

[17] Chen Y., Chiu C., Cheng Y. (2010). Influences of operational conditions and geometric parameters on the stiffness of aerostatic journal bearings. Precis. Eng..

[18] Swanson E., Heshmat H., Walton J. (2002). Performance of a foil-magnetic hybrid bearing. J. Eng. Gas Turbines Power.

[19] Cui H., Wang Y., Yue X., Huang M., Wang W. (2017). Effects of manufacturing errors on the static characteristics of aerostatic journal bearings with porous restrictor. Tribol. Int..

[20] Cui H., Wang Y., Yue X., Huang M., Wang W., Jiang Z. (2018). Numerical analysis and experimental investigation into the effects of manufacturing errors on the running accuracy of the aerostatic porous spindle. Tribol. Int..

[21] Zhang G., Zheng J., Yu H., Zhao R., Shi W., Wang J. (2021). Rotation Accuracy Analysis of Aerostatic Spindle Considering Shaft’s Roundness and Cylindricity. Appl. Sci..

[22] Zhang G., Zheng J., Yu H., Chen T., Zhang K., Dou G. (2022). Evaluation of the Influence of Shaft Shape Errors on the Rotation Accuracy of Aerostatic Spindle—Part 1: Modeling. Electronics.

[23] San Andrés L., Cable T.A., Zheng Y., De Santiago O., Devitt D. Assessment of porous type gas bearings: Measurements of bearing performance and rotor vibrations. Proceedings of the Turbo Expo: Power for Land, Sea, and Air. American Society of Mechanical Engineers.

[24] Liu W., Feng K., Huo Y., Guo Z. (2018). Measurements of the rotordynamic response of a rotor supported on porous type gas bearing. J. Eng. Gas Turbines Power.

[25] Zhang S., To S., Wang H. (2013). A theoretical and experimental investigation into five-DOF dynamic characteristics of an aerostatic bearing spindle in ultra-precision diamond turning. Int. J. Mach. Tools Manuf..

[26] Liu J. (2013). The Study of the Vibration Characteristics of Rotor-Gas Bearing System. Master’s Thesis.

[27] Cao Z. (2010). Study on the Static and Dynamic Characteristicsof Self-Acting Gas Bearing. Master’s Thesis.

[28] Zhang G. (2010). Research of Dynamic Characteristics for High Speed Hybrid Gas Bearing Rotor System. Ph.D. Thesis.

[29] Ansys Fluent. https://www.ansys.com/zh-cn/products/fluids/ansys-fluent.

